# Early Postoperative Results after Removal of Cranially Migrated Lumbar Disc Prolapse: Retrospective Comparison of Three Different Surgical Strategies

**DOI:** 10.1155/2014/702163

**Published:** 2014-11-13

**Authors:** C. Schulz, U. Kunz, U. M. Mauer, R. Mathieu

**Affiliations:** Department of Neurosurgery, German Federal Armed Forces Military Hospital, Oberer Eselsberg 40, 89081 Ulm, Germany

## Abstract

*Background*. To compare the early postoperative results of three surgical approaches to lumbar disc herniations that migrated cranially. Minimally invasive techniques such as the translaminar and endoscopic transforaminal approaches are utilized in patients with lumbar disc herniations to gain access to cranially located disc material and to avoid the potentially destabilizing resection of ligament and bone tissue, which is associated with an extended interlaminar approach. *Methods*. This retrospective study compares the postoperative pain and functional capacity levels of 69 patients who underwent an interlaminar (Group A, *n* = 27), a translaminar (Group B, *n* = 22), or an endoscopic transforaminal procedure (Group C, *n* = 20). *Results*. Median VAS scores for leg pain decreased significantly from before to after surgery in all groups. Surgical revisions were required in thirteen cases (five in Group A, one in Group B, and seven in Group C; *P* = 0.031). After six weeks, there were significant differences in back pain and functional outcome scores and in the results for the MacNab criteria but not in leg pain scores. *Conclusions*. The interlaminar and translaminar techniques were the safest and fastest ways of gaining access to cranially migrated disc material and the most effective approaches over a period of six weeks.

## 1. Introduction

Craniolateral lumbar disc herniations account for approximately 10% of all lumbar disc herniations [1]. The standard surgical procedure for this type of disc herniation involves an interlaminar approach. Disc herniations at this location (medial to the pedicle) require a craniolateral enlargement of the fenestration or even a hemilaminectomy and partial facetectomy [2]. This procedure may involve the removal of a major portion of the pars interarticularis and the facet joint [3]. Even a microsurgical interlaminar approach may require the resection of more than 50% of the facet joint in approximately two thirds of cases [4]. Such an extent of facet resection is believed to cause postoperative instability and postoperative local pain syndromes in the lumbar spine [1, 5, 6]. Less invasive approaches with preservation of the facet joint and the pars interarticularis have been developed for this special type of disc herniation. Apart from a translaminar approach (through the hemilamina) [7], an endoscopic transforaminal approach (through the neural foramen) can be utilized [8]. There are to date no studies investigating whether these two techniques are as effective and safe as the traditional interlaminar approach.

## 2. Materials and Methods

This noninterventional and nonrandomized observational study included 69 consecutive patients who were retrospectively assigned to one of three groups. Group A (*n* = 27) consisted of patients who underwent interlaminar surgery. Patients in Group B (*n* = 22) underwent a translaminar procedure. Group C (*n* = 20) were patients who underwent endoscopic transforaminal surgery. Included were patients who underwent sequestrectomy after isolated unilateral disc herniation and monoradiculopathy that was definitely correlated with disc herniation. Excluded were patients who underwent additional discectomy as well as patients with multiple segment involvement, polyradicular and bilateral symptoms, or associated spinal stenosis. Also excluded were patients with a history of spinal surgery or spinal trauma. All the included cases showed an extreme far-upward migration of the fragment higher than zone 1 (according to the classification of Lee et al., 2007; [8]). No fragment was located below the inferior margin or above the superior margin of the pedicle. The maximum size of the herniation was measured in the presurgical MR images (axial, sagittal, and coronal sections) and showed no statistical difference between the groups. The three surgical techniques used were performed according to the descriptions by Javedan et al. (interlaminar approach [9]), Schulz et al. (translaminar approach [10]), and Ruetten et al. (endoscopic transforaminal approach [11]). All surgeries were performed under general anaesthesia in a prone position. For the inter- and translaminar approach a midline incision of 2–4 cm was used, and the surgery was done in a microsurgical manner using the surgical microscope. For the endoscopic transforaminal approach however one small incision of no more than 1 cm (to insert the tubes for endoscopy, max. diameter 8 mm) was used. The placement of the incision and the movements of the working cannulas were performed under the control of biaxial fluoroscopy and for the sequestrectomy a uniportal full-endoscopic system was used. In all included cases only a sequestrectomy of the migrated fragments was performed. Cases with an additionally required discectomy (regardless of the reason) were excluded. All patients were on the same care paths and postoperative rehabilitation and return to work/daily life activity protocols.

Before surgery, at discharge from inpatient care and six weeks after surgery, outcomes were assessed using a visual analog scale (VAS) for back pain and leg pain, pain and work scales for general pain and functional capacity levels (according to Denis et al. [12]), and the MacNab criteria for patient satisfaction with the surgical result [13]. ASA scores [14], operating times, intra- and perioperative complication rates (root lesions, intraspinal hematoma, and CSF leakages), the number of postoperative revisions, and the length of hospital stay were compared.

The Friedman test was used to detect differences in VAS scores within groups at the different time points (dependent samples). The chi-squared test was used to analyze pain and work scale scores and the results for the MacNab criteria between groups at the same time point. An analysis of variance (ANOVA) was used to compare continuous data and the chi-squared test to compare nominal data. The level of significance was set at *P* < 0.05. Statistical analyses were performed using SPSS Statistics 21.0.

## 3. Results

Table 1 provides an overview of preoperative patient data. There were no significant differences between the groups except for the number of patients with preoperative paresis. The procedures were performed by surgeons with varying levels of qualification, that is, either a specialist or a resident under the guidance of a specialist. (A specialist in this study was defined as a spine surgeon after his/her board examination that requires a minimum surgical education of 6 years. Surgeons with a shorter time of education were defined as residents.) This distribution shows inhomogeneity, however, just failed significance level as indicated by a *P* value greater than 0.05.

Table 2 shows the duration of surgery, the length of hospital stay, the rates of perioperative surgical complications, and the number of surgical revisions. Operating times were significantly longer for Groups A and C than for Group B. During surgery, root lesions occurred in three cases (all in Group C). Epidural bleeding was noted in one case in Group A. No CSF leakages occurred. The difference in the rate of this perioperative surgical complication was just not significant (0.097, chi-squared test). Group A and C patients required a significantly higher number of surgical revisions than Group B patients (0.031, chi-squared test). None of the revision cases in Groups A (*n* = 5) and B (*n* = 1) occurred within the first two weeks after surgery. By contrast, 6 of the 7 patients in Group C required revision surgery within the first two weeks after primary surgery. The length of the inpatient care did not show significant differences.

Table 3 provides an overview of the pain and functional outcome scores and the results for the MacNab criteria that were obtained at the various time points. The three groups of patients showed a significant decrease in all clinical parameters from the preoperative assessment to the six-week follow-up evaluation. At the time of discharge from inpatient care, VAS back and leg pain scores and the results for the MacNab criteria were significantly different from those obtained before surgery. By contrast, there was no significant difference in pain scale scores (according to the Denis classification). Whereas there were no significant differences in VAS leg pain and pain scale scores between the preoperative and the six-week follow-up evaluation, the difference in VAS back pain scores continued to be significantly different. In addition, there were significant differences between the groups in functional outcome (work scale) and patient satisfaction (MacNab criteria) after six weeks.

## 4. Discussion

Approximately 90% of patients who underwent microsurgical interlaminar removal of craniolaterally displaced disc fragments were found to have good or excellent postoperative results as assessed by MacNab criteria [15]. The same good results were also reported after translaminar sequestrectomy [16–20]. Our Group A and B patients showed similar results. The endoscopic transforaminal extirpation of free disc fragments within the lumbar canal (not in the region of the foramen and not cranially migrated) is also associated with good and excellent results in approximately 90% of the cases [11]. Such a success rate, however, will not be achieved in the case of far craniolateral sequestrated disc fragments located medial to the pedicle. This is supported by Lee et al., showing that the endoscopic transforaminal approach is not well suited for patients with disc herniations in this location as indicated by longer operating times, higher intraoperative complication rates, and higher revision rates [8]. The farther a disc fragment migrates in the cranial direction, the worse the results are. A safe transaxillary inspection of the ventral epidural space is even more technically demanding in patients with a far cranial disc fragment and additional narrowing of the spinal canal and/or foramen [8, 21]. This area is difficult to visualize and is what was aptly termed a “hidden zone” by MacNab [13]. Even when the superior articular process and the lower edge of the superior pedicle are partially resected with an endoscopic approach to allow a cranial enlargement of the foramen and the cranial retraction of the nerve root for easier manipulation, the relevant area cannot be completely visualized using the transforaminal approach [22]. The most important results in this analysis are the decreased safety, the prolonged duration of the surgery, and the increased rate of surgical revision for symptomatic herniations in the group after endoscopic transforaminal surgery. It is more than questionable in our opinion whether the endoscopic transforaminal approach is appropriate for extremely cranially migrated disc herniations located medial to the pedicle. When endoscopic techniques are used, if at all, for this special type of disc fragment, interlaminar or translaminar techniques appear to be best suited since they are reported to yield surgical outcomes comparable to those following open interlaminar procedures [23–26].

The clinical results after translaminar sequestrectomy are better than those following endoscopic transforaminal surgery and tend to be better than those after an interlaminar approach in our study. But the clinical relevance of differences in outcome as measured by VAS and Denis Scales, although some were proven to be statistically significant, may be questionable. The differences in the MacNab criteria however demonstrate a significant lower number of satisfied patients in the endoscopic cohort. The differences in clinical outcome between the inter- and the translaminar group are not significant. Additionally the perioperative complication as well as postoperative revision rate did not show significant differences during a six-week interval. The only significant difference found was the duration of surgery. We conclude that considering the early postoperative results there is no rationale to prefer the translaminar over the interlaminar technique. Indeed, we were able to perform smaller skin incisions and recorded a shorter duration of surgery in most of the translaminar cases, but this did not result in significant better clinical results over interlaminar cases. So the clinical relevance of the size of skin incisions (2 or 4 cm) and surgery duration (meaning 74 min. versus 57.5 min) remains questionable. Using the translaminar approach, the sparing of the pars interarticularis and the facet joint can be easier provided than in the interlaminar approach. But after only 6 weeks and without functional imaging, we are not able to unequivocally show a relevant advantage for the translaminar approach. For this issue a longer observation period in a prospective setting including radiographic examination of segmental instability is necessary. In the retrospective analysed cases presented here, however, functional imaging was not performed during the observation period. Postacchini et al. conducted a study in which they followed up 43 patients who underwent surgery for intraforaminal or extraforaminal herniations using an interlaminar approach [4]. At a two-year followup, the 29 patients (67%) with a facetectomy of more than 50% did not show a higher rate of instability or local pain than the 14 patients with a facetectomy of 50% or less. In spite of the small number of patients in the comparative group, the study suggests that the role of unilateral partial or subtotal lumbar facetectomy appears to be overestimated in terms of postoperative instability and pain reduction at least in the short term.

The strength of our study is limited by the lack of randomization, the lack of blinding, and the small number of patients in each group. For the future, prospective data in a powerful number of cases per group and an independent reexamination after an interval of minimum 6 months postoperatively have to be collected. In addition, we assume a relevant bias resulting from the inhomogeneous distribution of surgeons with different levels of experience and expertise in the three groups (despite the absence of statistical significance). There was additionally a significant difference in the number of paretic patients before surgery, with far fewer cases in the endoscopic cohort. Such differences could affect outcome and one would expect a higher rate of worse results in the inter- or translaminar group. But actually the better neurological precondition in the endoscopic group did not result in a better clinical outcome in this cohort. Prospective randomized studies with larger numbers of patients and longer follow-up periods are needed in which the competing methods (interlaminar versus translaminar versus paraspinal oblique versus endoscopic translaminar versus endoscopic interlaminar) are assessed and compared.

## 5. Conclusions

All three surgical approaches led to a significant reduction of preoperative pain. The interlaminar and translaminar techniques were the safest and fastest ways of gaining access to cranially migrated disc material and also proved to be the most effective approaches over a period of six weeks and the most successful in relieving pain. The endoscopic transforaminal approach was relatively unsuitable for the removal of cranially displaced disc fragments. By contrast, endoscopic translaminar and endoscopic interlaminar approaches may lead to better results in the future.

## Figures and Tables

**Table 1:** Preoperative patient data.

	Group A (interlaminar approach) *n* = 27	Group B (translaminar approach) *n* = 22	Group C (endoscopic transforaminal approach) *n* = 20	Level of significance
Gender (females/males)	9/18	8/14	5/15	0.717 chi^2^

Age (years) MED (MIN–MAX; SD)	59 (38–85; 15)	59 (37–76; 10)	58 (28–79; 13)	0.592 ANOVA

Preoperative ASA score	I: 2; II: 10; III: 11; IV: 4; V: 0; VI: 0	I: 3; II: 8; III: 10; IV: 1; V: 0; VI: 0	I: 2; II: 8; III: 7; IV: 3; V: 0; VI: 0	0.903 chi^2^

Operated level	L1/2: 0; L2/3: 1; L3/4: 8; L4/5: 11; L5/S1: 7	L1/2: 2; L2/3: 3; L3/4: 5; L4/5: 7; L5/S1: 5	L1/2: 1; L2/3: 1; L3/4: 6; L4/5: 11; L5/S1: 1	0.365 chi^2^

Preoperative paresis	23/27 (85.2%)	18/22 (81.8%)	10/20 (50%)	**0.015** **c** **h** **i** ^2^

Preoperative sensory deficit	18/27 (66.7%)	19/22 (86.4%)	15/20 (75%)	0.282 chi^2^

Surgeon (specialist/resident)	20/7	18/4	20/0	0.053 chi^2^

MED: median.

MIN: minimum.

MAX: maximum.

SD: standard deviation.

Chi^2^: chi-squared test.

ANOVA: analysis of variance.

ASA: American Society of Anesthesiologists.

**Table 2:** Surgical results.

	Group A (interlaminar approach) *n* = 27	Group B (translaminar approach) *n* = 22	Group C (endoscopic transforaminal approach) *n* = 20	Level of significance
Duration of surgery (minutes) MED (MIN–MAX; SD)	74 (35–140; 28.13)	57.5 (38–75; 11.98)	105 (60–180; 34.9)	**<0.001** **ANOVA**

Surgical revisions for symptomatic herniation	5/27 (14.8%)	1/22 (4.5%)	7/20 (35%)	**0.031** **c** **h** **i** ^2^

perioperative surgical complications	1/27 (3.7%)	0/22 (0%)	3/20 (15%)	0.097 chi^2^

Length of hospital stay (days) MED (MIN–MAX; SD)	8 (7–30; 5)	8 (6–13; 1)	9 (6–22; 5)	0.076 ANOVA

MED: median.

MIN: minimum.

MAX: maximum.

SD: standard deviation.

ANOVA: analysis of variance.

Chi^2^: chi-squared test.

**Table 3:** Clinical scores at different time points.

Clinical score	Time point	Group A (interlaminar approach) *n* = 27	Group B (translaminar approach) *n* = 22	Group C (endoscopic transforaminal approach) *n* = 20	Level of significance
VAS back pain MED (MIN–MAX; SD)	Before surgery	9 (6–10; 0.94)	8 (7–10; 0.74)	8.5 (7–10; 0.69)	0.905; KWT
	At discharge	4 (2–5; 0.94)	3 (1–5; 0.91)	4 (3–6; 0.99)	**<0.001; KWT**
	Six weeks after surgery	2 (1–4; 0.72)	1.5 (1–4; 0.73)	3 (1–5; 0.93)	**<0.001; KWT**

		**<0.001; FT**	**<0.001; FT**	**<0.001; FT**	

VAS leg pain MED (MIN–MAX; SD)	Before surgery	7 (4–9; 1.5)	7 (5–9; 1.14)	7.5 (6–9; 1)	0.130; KWT
	At discharge	3 (2–5; 0.75)	3 (2–4; 0.73)	3.5 (2–5; 0.89)	**0.021; KWT**
	Six weeks after surgery	2 (1–3; 0.7)	2 (1–3; 0.73)	2 (1–3; 0.64)	0.190; KWT

		**<0.001; FT**	**<0.001; FT**	**<0.001; FT**	

Denis pain scale (I to V)	Before surgery	I: 0; II: 0; III: 0; IV: 6; V: 21	I: 0; II: 0; III: 0; IV: 5; V: 17	I: 0; II: 0; III: 0; IV: 5; V: 15	0.974; chi^2^
	At discharge	I: 0; II: 0; III: 7; IV: 20; V: 0	I: 0; II: 1; III: 10; IV: 11; V: 0	I: 0; II: 0; III: 5; IV: 13; V: 2	0.124; chi^2^
	Six weeks after surgery	I: 5; II: 9; III: 12; IV: 1; V: 0	I: 5; II: 8; III: 9; IV: 0; V: 0	I: 2; II: 6; III: 9; IV: 3; V: 0	0.471; chi^2^

		**<0.001; ** **c** **h** **i** ^2^	**<0.001; ** **c** **h** **i** ^2^	**<0.001; ** **c** **h** **i** ^2^	

Denis work scale (I to V)	Before surgery	I: 0; II: 0; III: 0; IV: 10; V: 17	I: 0; II: 0; III: 0; IV: 8; V: 14	I: 0; II: 0; III: 0; IV: 11; V: 9	0.378; chi^2^
	Six weeks after surgery	I: 0; II: 10; III: 17; IV: 0; V: 0	I: 0; II: 13; III: 9; IV: 0; V: 0	I: 0; II: 3; III: 15; IV: 2; V: 0	**0.014; ** **c** **h** **i** ^2^

		**<0.001; ** **c** **h** **i** ^2^	**<0.001; ** **c** **h** **i** ^2^	**<0.001; ** **c** **h** **i** ^2^	

MacNab criteria (I to IV)	At discharge	I: 15; II: 12; III: 0; IV: 0	I: 13; II: 9; III: 0; IV: 0	I: 2; II: 13; III: 4; IV: 1	**0.002; ** **c** **h** **i** ^2^
	Six weeks after surgery	I: 6; II: 17; III: 4; IV: 0	I: 10, II: 11, III: 1, IV: 0	I: 0; II: 4; III: 11; IV: 5	**<0.001; ** **c** **h** **i** ^2^

		**<0.001; ** **c** **h** **i** ^2^	**<0.001; ** **c** **h** **i** ^2^	**<0.001; ** **c** **h** **i** ^2^	

VAS: visual analog scale.

MED: median.

MIN: minimum.

MAX: maximum.

SD: standard deviation.

KWT: Kruskal-Wallis test.

FT: Friedman test.

chi^2^: chi-squared test.

MacNab criteria I (excellent), II (good), III (fair), and IV (poor).
